# Ozonated Sunflower Oil Stimulates the Local Antioxidant System and Helps Meglumine Antimoniate to Ameliorate Cutaneous Leishmaniasis Lesions

**DOI:** 10.1021/acsomega.4c11263

**Published:** 2025-04-22

**Authors:** Isaac Loreiro Cabral, Lucas Bonatto de Souza Lima, Daniela Patrícia Três, Carla Diel Fabrini, Gislayni Carolini da Silva, Camilla Zottesso Pellon Ferreira, Fernanda Coleraus Silva, João Paulo de Arruda Amorim, Thaís Soprani Ayala, Rafael Andrade Menolli

**Affiliations:** † Laboratory of Applied Immunology, Center of Medical and Pharmaceutical Sciences, Western Parana State University, 2069 Universitaria st, Jd. Universitario, 85819-110, Cascavel, PR, Brazil; ‡ Center of Biological and Health Sciences, Western Parana State University, 2069 Universitaria st, Jd. Universitario, 85819-110, Cascavel, PR, Brazil

## Abstract

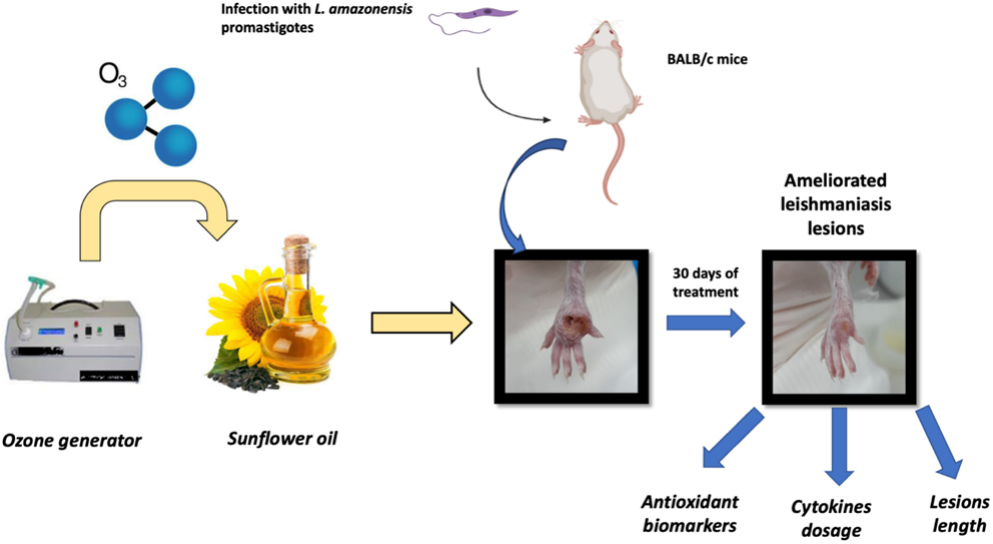

Cutaneous leishmaniasis is an infectious disease that causes disfiguring scars, which are not eliminated by drugs available to the treatment. Besides, these marks lead to economic and social and economic losses. Ozonated oil has wound healing activity as proven in the literature, and its use with the standard treatment to leishmaniasis could enhance the therapy. This study investigated ozonated sunflower oil as an adjuvant to meglumine antimoniate (MA) in the treatment of lesions caused by *Leishmania amazonensis*. BALB/c mice were infected with the parasite, and after the lesions appeared, they were subjected to different schedules through standard drug treatment (MA) with or without ozonated oil. After one month of the treatment, were evaluated the lesions thickness and their parasite burden; besides, the production of nitric oxide and cytokines from draining lymph node cells and peritoneal macrophages were determined. ERK1/2 expression and the concentrations of mature collagen and antioxidant enzymes were evaluated in the treated paws. The group that received MA with ozonated oil presented the best results, ameliorating the lesion, as shown by the macroscopic aspects and quantity of the mature collagent. The parasites were eliminated from the lesion, showing that ozonated oil enhanced the leishmanicidal action of MA. The reduction in lesions can be partially attributed to the stimulation of the local antioxidant system, which reached significantly greater levels in the MA plus topical ozonated oil group than MA and nontreated groups. So, the treatment of the experimental cutaneous leishmaniais with ozonated sunflower oil as adjuvant proved to be effective, increasing the leishmanicidal and wound healing effects of meglumine antimoniate.

## Introduction

The World Health Organization (WHO) classifies leishmaniasis as neglected tropical diseases (NTDs). These parasitic diseases are more frequent in the poorest regions of the globe, with about 350 million people at risk of infection.^1^ Protozoa of the genus *Leishmania* cause this disease, which are transmitted by the bite of sand flies, when infected females feed on the blood of a mammalian host.^2^

Leishmaniasis can present various clinical forms, including cutaneous, mucocutaneous, and visceral. The distinct forms are mainly caused by differences in host immunities and diverse parasite species. Cutaneous leishmaniasis (CL) form is the most prevalent, showing around 800,000 new cases/year. Most of the cases are in the Americas, the Mediterranean area, the Middle East, and Central Asia; once, it is a disease linked to poverty.^3^

Although death by cutaneous leishmaniasis is rare, the scars after infection are often disfiguring and cause social and economic losses to the patient.^4^ Drug therapy is essential for accelerating healing in CL treatment, reducing fibrosis constitution, preventing evolution to aggressive forms, and preventing infection of other people.^5^ Currently, there are few available drugs, and the group of pentavalent antimonials, including meglumine antimoniate, are the most commonly used in CL. Nevertheless, they have proven adverse effects, and a prolonged period of use can cause parasite resistance.^6^

Alternative strategies of treatment, using topical substances, have been used in the intent to control CL,^7^ and ozonated oil is already used topically for the treatment of chronic wounds, leading to a reduction in the time of recovery and increasing treatment adherence, among other advantages.^8,9^ Therefore, we investigated the performance of ozonated sunflower oil as an adjuvant to meglumine antimoniate in lesions of experimental CL.

## Results

### Ozone Levels

Ozone becomes stable when bubbled into oils, so the ozone levels in the ozonated sunflower oil used here remained similar from days 0 to 30 (Supporting Figure 1).

The ozone concentration (indirectly determined by Pv) was significantly greater in the samples obtained from ozonated oil than in those obtained from nonozonated oil, reaching values that were more than five times greater.

### Paw Thickness and Aspects of the Lesions

**Figure 1:**
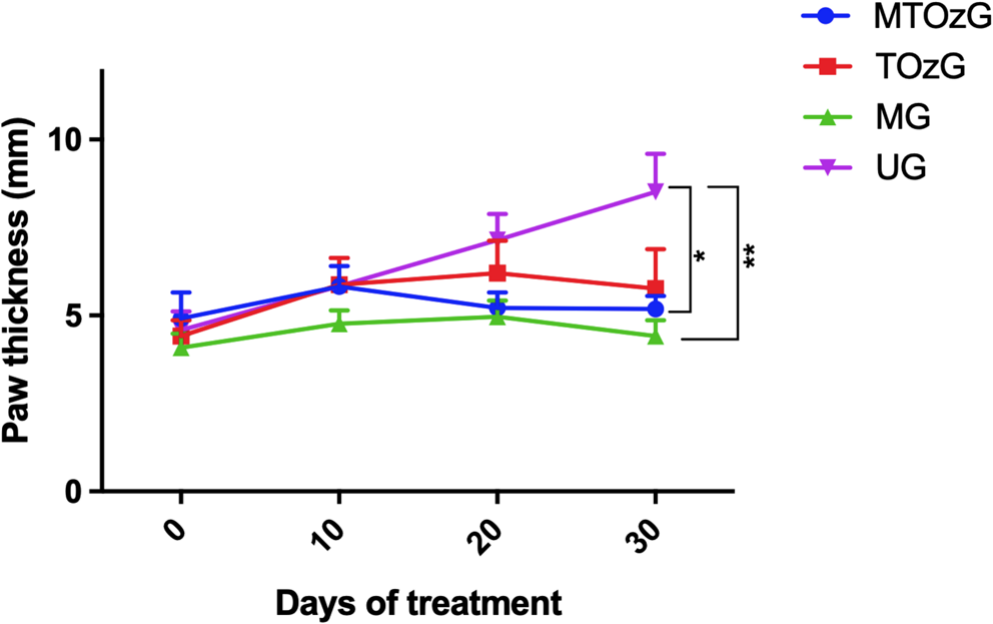
Measurement of mice paws infected with *L. amazonensis* and followed for 30 days of treatment: MTOzG, Meglumine antimoniate IP and topical ozonated oil group; TOzG, Topical ozonated oil group; MG, Meglumine antimoniate I.P. group; and UG, Untreated group. The values are the mean ± SEM of four to six animals per group. *Significantly different (*p < 0.05).

Figure 1 shows the paw thicknesses of infected animals during the treatment period with ozonated oil and MA (30 days). As expected, the group that did not receive treatment (U) exhibited continuous paw thickness growth, whereas those that received treatment maintained their paw size.

Compared with that of the untreated group (UG), the efficacy of the MA group (MG) alone or in combination with the topical ozonated oil group (MTOzG) was apparent with almost total closure of the lesions in all animals. The diminishment of paw thickness was significant (*p* < 0.05). These two groups increased the paw thickness by 8.08% (±2.45) and 5.45% (±15.1), whereas the topical ozonated oil group (TOzG) increased the paw thickness by 30.50% (±30.25), which, despite not reaching a significant difference from the untreated group, did not exacerbate these lesions.

One of the main aspects of cutaneous leishmaniasis is the disfiguring scars in patients affected by this disease. Thus, although MTOzG did not reduce the thickness of paws differently from the MG, the lesions aspects of the former are better (Supporting Figure 2) than those of the latter.

### Parasite Burden

The paw segments destined for culture on 199 medium (Table 1) revealed that the number of parasites in the groups that received MA (including or not ozonated oil) after 10 days of incubation was significantly lower than that in the groups without the drug. These findings indicated that ozonated oil acted as an adjuvant for lesion resolution but did not interfere with parasite development.

**Table 1:** Titers of Culturing of Fragments from *L. amazonensis*-Infected Paws on 199 Medium^a^

days after inoculum				
groups	1	2	3	4
MTOzG	NF	NF	NF	1/3
TOzG	1/192	1/192	1/192	1/384
MG	NF	NF	NF	1/3
UG	1/384	1/384	1/384	1/384

a MTOzG (meglumine antimoniate IP and topical ozonated oil group); TOzG (topical ozonated oil group); MG (meglumine antimoniate IP group); UG ( Untreated group). The values are the means of four to six experimental units per group. The values are presented as the inverse of the titers from the culture dilutions (as explained in the Methods section). *NF (not find—no promastigotes were found).

### Effects of Treatment on ERK 1/2 Expression in Infected Paws

The different aspects of lesions in the treatments were investigated by WB analysis to look at the intracellular pathway altered by the glucantime, glucantime plus ozonated oil, or only ozonated oil.

**Figure 2:**
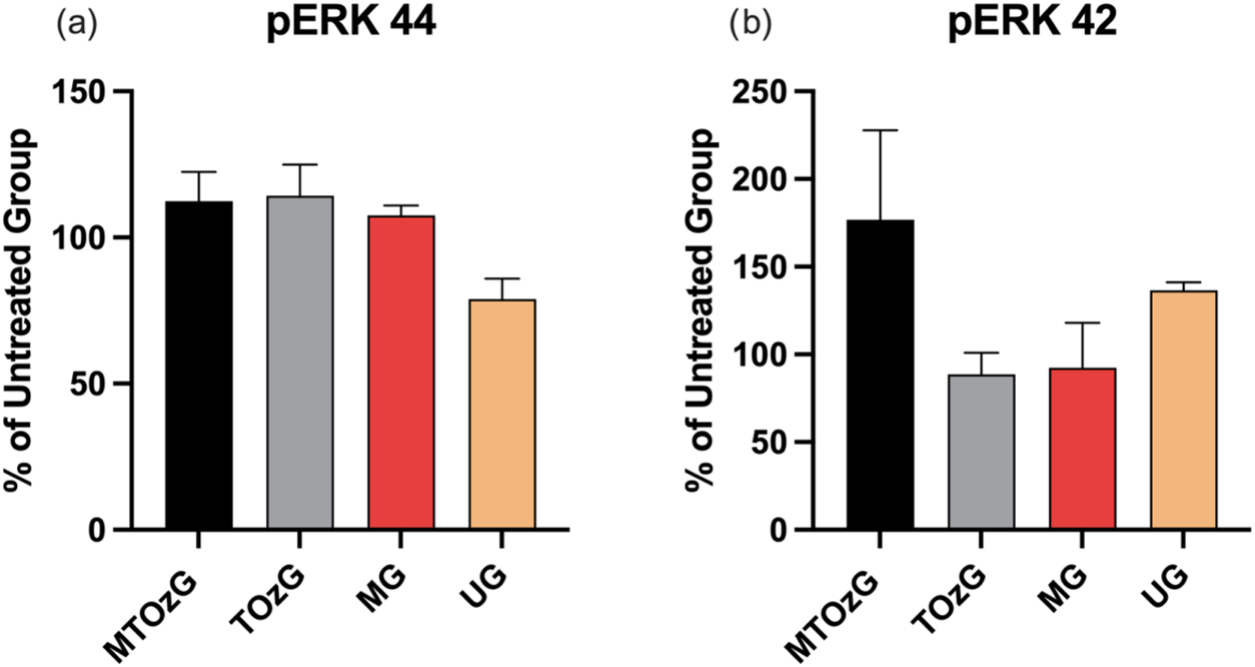
Analysis of the MAPK signaling pathway in *L. amazonensis-*infected paw homogenates after 30 days of treatment. (a) Phospho-ERK 44/GAPDH ratio (adjusted to the untreated group (UG) as 100%). (b) Phospho-ERK 42/GAPDH ratio (adjusted to the untreated group (UG) as 100%). The data are presented as the means of three measurements (±SEM). Any significant differences were observed between the groups.

Figure 2 shows the intensity of the phosphorylated intermediates ERK 1 and ERK 2 (44 and 42 kDa, respectively) from the tissue of infected paws. Although the differences in ERK 1 expression in the tissues of the different treatment groups were not significant (Figure 2a), ERK 1 expression in the untreated group was similar in the treated groups.

The detection of ERK 2 showed a similar situation, with no significant difference compared with the other groups, but the tissue from the paws treated with MA plus ozonated oil reached a greater level.

### Tissue Antioxidant Activity

**Figure 3:**
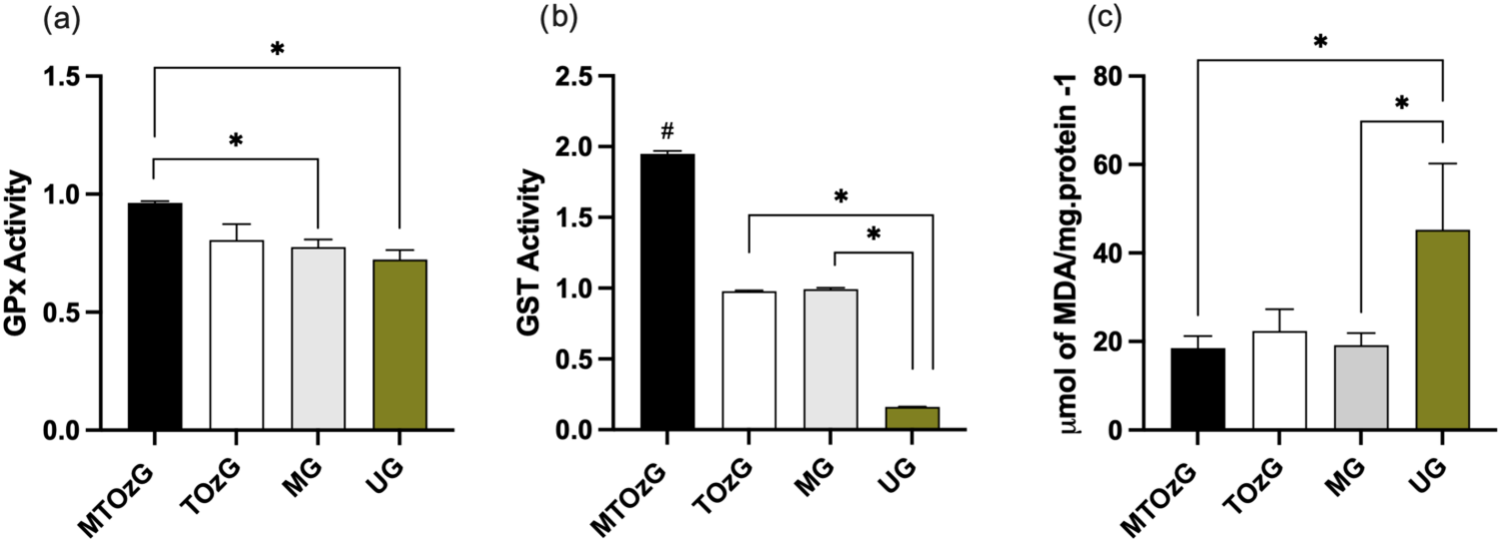
Biomarkers of oxidative stress in paws from BALB/c mice infected with *L. amazonensis* after treatment for 30 days of treatment. MTOzG—Meglumine antimoniate IP and topical ozonated oil group; TOzG—Topical ozonated oil group; MG—Meglumine antimoniate IP group; UG—Untreated group. (a) GPx, glutathione peroxidase activity; (b) GST, glutathione S-transferase activity; (c) Lipid peroxidation (calculated from an MDA curve, with results expression in μmol of MDA/mg of protein). The data are presented as the means of three experiments performed in triplicate (±SEM) with segments of the paws from four to six animals per group. #, different from the other groups, # and *p < 0.05.

The antioxidant activities of GPx and GST are shown in Figure 3. Both results (a and b) demonstrated that the group that received both treatments (MA and topical ozonated oil) presented greater antioxidant activity in the tissues, with significant differences from MG, the other group that resolved the lesion, and UG, the nontreated group. In the GPx analysis, both groups that received topical ozonated treatment did not differ, but a statistical difference was observed in the GST.

The measurement of lipoperoxidation in the paws was significantly greater in the nontreated animals than in the animals treated with meglumine antimoniate or meglumine plus ozonated oil. Furthermore, the group treated with only ozonated oil topically presented lower levels than did the nontreated group, but the difference was not significant (Figure 3c).

### Mature Collagen Detection

**Figure 4:**
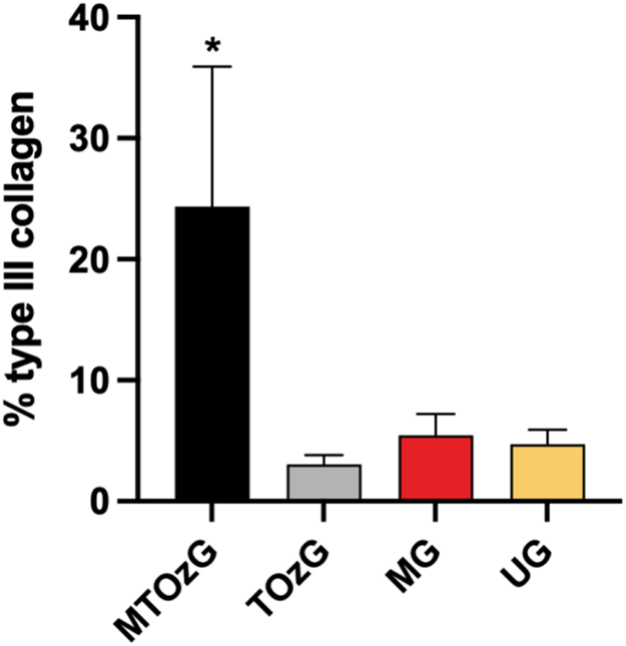
Analysis of the concentration of collagen type I in histological sections stained with picrosirius red from lesions of the paws of mice infected with *L. amazonensis*. The data are presented as percentages of collagen in the analyzed tissue. MTOzG—Meglumine antimoniate IP and topical ozonated oil group; TOzG—Topical ozonated oil group; MG—Meglumine antimoniate IP group, and UG—Untreated group. The values shown are the mean ± SEM of five slides from each animal (four to six animals per group). *Significantly different (*p < 0.05).

Microscopic analysis of sections stained with picrosirius red staining revealed that the lesions from the MTOzG group had a greater percentage of type I collagen fibers (Figure 4) than those in the other groups, demonstrating an advanced state of wound healing; once type I collagen is a more resistant fiber, the proliferative stage of cicatrization is established. A panel with representative images of picrosirius red staining of all of the groups is shown in Supporting Figure 3.

### Systemic and Local Effects on the Immune System

Cellular aspects and cytokine secretion associated with systemic modifications of the immune system are detected in infected animals. Differential counting of total leukocytes revealed that the percentages of lymphocytes and neutrophils in MTOzG did not significantly differ between the beginning and end of the treatment. However, the other groups presented a greater number of lymphocytes on the first day, and this percentage decreased significantly over the study period. The number of neutrophils reversed but increased over time (Supporting Figure 4).

**Figure 5:**
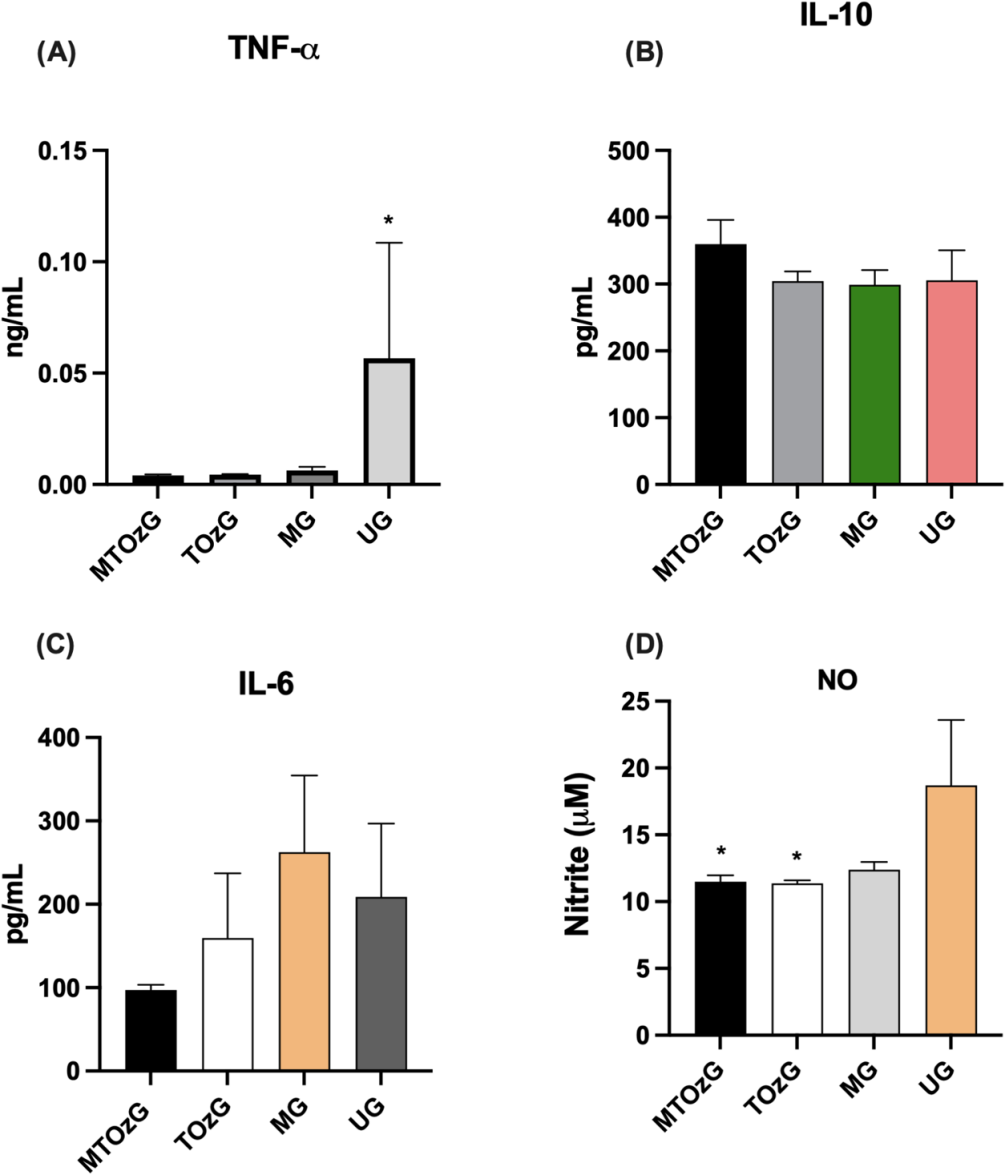
(a) TNF-α, (b) IL-10, (c) IL-6, and (d) NO secretion by peritoneal macrophages from animals with cutaneous experimental leishmaniasis after treating for 30 days. MTOzG—Meglumine antimoniate IP and topical ozonated oil group; TOzG—Topical ozonated oil group; MG—Meglumine antimoniate IP group, and UG—Untreated group. The values shown are the mean ± SEM of four to six animals per group. *Significantly different (*p < 0.05).

Although the behavior of blood leukocytes was similar in the TOzG, MG, and UG groups, cytokine and NO secretion by peritoneal macrophages demonstrated a pronounced inflammatory effect with TNF-α reaching higher concentrations in the untreated group (UG) than in the treated groups. In contrast, the levels of IL-10, an anti-inflammatory cytokine, did not differ (Figure 5A,B). Confirming this finding, NO secretion by peritoneal macrophages from animals that did not receive treatment reached a greater level than that of the other animals without a significant difference from that of the MG (meglumine group) (Figure 5D). IL-6 secretion was lower in the MTOz group than in the other groups, but the difference was not significant (Figure 5C).

**Figure 6:**
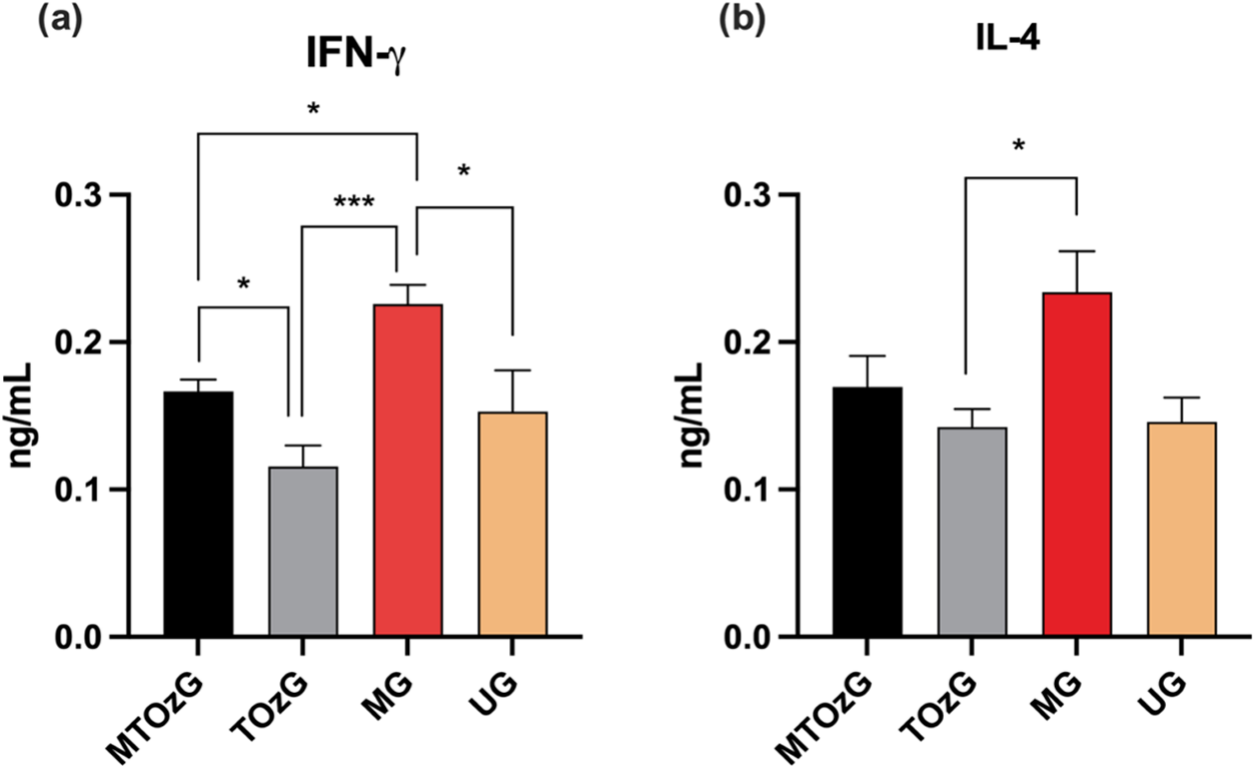
IFN-γ (a) and IL-4 (b) levels (ng/mL) in the supernatants of total cells obtained from the draining lymph nodes of *L. amazonensis-*infected BALB/c animal paws after 30 days of treatment. The values represent the mean ± SEM of six experimental units per group. MTOzG—Meglumine antimoniate IP and topical ozonated oil group; TOzG—Topical ozonated oil group; MG—Meglumine antimoniate IP group, and UG—Untreated group. *Significant difference (*p* < 0.05).

Local cytokine production revealed that in the MG (which received only MA), the lymph node cells reached a higher activation level and produced significantly more IFN-γ and IL-4 than the other groups did. The MA can stimulate leukocytes,^10^ which explains this increased concentration, as parasite elimination was similar in the other group that received MA but with topical ozonated oil (MTOzG). Nevertheless, ozonated oil can modulate the immune system locally, reducing the level of production of cytokines. These data are shown in Figure 6A,B.

## Discussion

The use of sunflower ozonated oil as an adjunct to MA reduced the lesion extension of *L. amazonensis-*infected animals, resulting in better recovery than when MA was used alone. However, ozonated oil alone did not provide this regression. Ozone can accelerate wound healing,^11^ and this characteristic should be utilized by adding ozonated oil to the arsenal of therapies against CL once it is inexpensive and accessible.

CL treatment is prolonged and painful, especially when MA, an injectable drug that requires several doses during therapy, is used. The lesions of CL provoke disfiguring scars, leading to social and economic impacts on patients, primarily those living in underdeveloped countries.^4,12^ Thus, in addition to parasite elimination, an ameliorated cicatrization is essential for CL patients and can be achieved via the use of adjuvants in current standard drug therapy.

This study demonstrated that the ozone concentration remained very high for the treatment period, maintaining their properties. Oil has already been demonstrated to be an excellent vehicle for ozone because, unlike water, which is inoculated as a gas, ozone is very unstable; in oily substances, ozone creates bridges with ozonide, remaining a source of components with biological activity for months.^13,14^

A poor effect was observed in the treatment with sunflower ozonated oil alone, which could be explained by the difficulty in penetrating more profoundly into the lesion tissue and attacking *L. amazonensis* inside the cell. Several studies related the antimicrobial characteristics of ozonated oils in bacterial^15,16^ and fungal infectious skin diseases,^17,18^ but all of these conditions are superficial and caused by extracellular microorganisms, which are not characteristic of Leishmania, an intracellular parasite.^19^

Cutaneous leishmaniasis causes substantial production of ROS, which causes inflammation through the production of proinflammatory intermediary. ROS produced throughout a response lead to oxidative injury in uninfected cell, causing liberation of free radicals that cause collagen damage.^20^ Furthermore, vigorous secretion of radicals from oxygen (O_2_^–^) impairs antioxidant system, leading to cellular lesion during *L. amazonensis* infection.^21^

In this study, the activation of glutathione peroxidase and glutathione transferase at relatively high levels, together with better paw healing, demonstrated the capacity of ozonated sunflower oil in this direction. Furthermore, ROS strongly activate mitogen-activated protein kinases (MAPKs), such as ERK1/2 and c-Jun N-terminal kinases (JNKs), leading to NF-κB nuclear translocation.^22^ However, we did not detect differences in phosphorylated ERK1/2 expression between the groups. Ozonated oil increases the activity of the cellular antioxidant machinery, resulting in reduced damage caused by ROS and providing better wound healing. The activation of the antioxidant-responsive element (ARE), mainly the Nrf2 (nuclear factor erythroid 2-related factor 2) pathway, is responsible for this action.^23^ The activation of NF-κB also occurs, leading to the production of TGF-β, which is involved in cicatrization.^24^

The animals treated with ozonated oil and MA presented a relatively high concentration of mature collagen after 30 days of treatment, indicating a better effect on the lesion at the end (Supporting Figures 2 and 3). Wound healing is a crucial event in the treatment of CL and can cause permanent scar formation after curing, leading to social stigmatization, psychiatric disorders, and decreased quality of life in CL patients.^32^ Accelerated and ordinated cicatrization contributes to minimal scarring when the treatment is finished with the CL, contributing to better adhesion to medicamentous therapy. In the CL, more immature collagen (type III) cooccurs with more local parasites, whereas elimination and cicatrization provide more type I collagen.^33^ However, in this study, when the parasite was eliminated by MA (group 3), the quantity of collagen produced was significantly lower than that produced by treatment with both MA and ozonated oil, indicating the efficacy of MA in eliminating *Leishmania* but not in tissue healing.^34^

The lymph node cells from the MA-treated group produced significantly more IFN-γ than those from the other groups did, which was attributed to almost total parasite elimination and the ability of the meglumine antimoniate to activate leukocytes.^10^ Nevertheless, the group treated with MA and ozonated oil did not reach the same level of interferon production, which could be an effect of the ozonated oil. IL-4 secretion was significantly greater in the MA-treated group (MG) than in the topical ozone group. However, all of the groups produced this cytokine, which is characteristic of *L. amazonensis* infection in BALB/c mice.^25^ The elimination of the *Leishmania* parasites requires a strong inflammatory immune reaction characterized by certain cytokines, such as IFN-γ and TNF-α, from the host. On the other hand, the alternative activation of macrophages, provoked by the IL-4, culminates in chronic infection and parasite viability.^26^

The joint therapy of ozonated sunflower oil and MA was effective in the treatment of the leishmaniasis cutaneous lesions in an animal model, causing eradication of *L. amazonensis* and promoting recovery of the paws in a treatment period of 30 days. Thus, the use of ozonated sunflower oil as an adjunct may provide a shorter treatment time for patients, who are consequently less exposed to the side effects of long-term standard treatment.

## Materials and Methods

### Parasite Culture

*Leishmania* (*Leishmania*) *amazonensis* (MHOM/BR/1977/LTB0016), in promastigote forms, were cultivated and kept via weekly transplants in 199 medium supplied with 10% fetal bovine serum and hemin (5 μg/mL).

### Animals

24 female BALB/c mice, 6–8 weeks old, received regular feed and water *ad libitum* and were maintained in common cage under a 12 h light–dark cycle. All procedures were approved by the Ethics Committee on the Use of Animals of UNIOESTE (n° 01/2021) and followed the Brazilian Law for Scientific Use of Animals (Law 11,794, 10/8/2008). Each animal in the cage received a mark, which was used to randomly select the mouse, setting up the treatment groups at random.

### Ozonated Oil Obtainment and Level Measurement

Commercial sunflower oil (Liza) received 10% distilled water and Tween 80 (0.5%) as emulsifying agents and was ozonated as described by Moureu et al., with modification.^27^ An ozone generator (Ozone & Life, mod. 1.5 RM) was used for 15 h, bubbling in the oil in an ozone output of 99 ppm. A sufficient volume of the same ozonated oil was used in all of the experiments.

The ozone levels were indirectly assessed, and the hydrogen peroxide (H_2_O_2_) levels in the oil were determined via a iodometric method following Martinez Tellez et al.^28^ To 0.5 g of oil was added a mixture of 18 mL of glacial acetic acid, 12 mL of chloroform, and 0.5 mL of potassium iodide (K.I.) saturated solution. In the sequence, the mixture was incubated in the dark overnight. Next, distilled water was added, and titration with 0.01 M sodium thiosulfate (Na_2_S_2_O_3_) was done, until the color disappeared. Finally, a starch mixture (5 mL) was inserted, and a new titration occurred. The following formula was used to obtain the peroxide values (Pv)
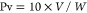
where *V* represents the sodium thiosulfate quantity in milliliters, and *W* represents the oil weight (g). The Pv concentration (mEq/kg) was converted to milligrams of ozone per gram of oil, following the methods of Skalska et al.^29^ An estimated ozone concentration in μg/mL of oil was subsequently determined considering the density of oils from 920 to 930 kg/m^3^.^30^ The ozone levels from sunflower oil used in the treatments were measured (in triplicate) every 10 days for one month to evaluate their stability. The concentration of Pv in the nonozonated oil was determined similarly.

### In Vivo Experimental Procedure

#### Lesion Development

A suspension of *L. amazonensis* (1 × 10^5^ parasites) in phosphate-buffered saline (PBS) was inoculated intradermically (0, 1 mL) into the plantar dorsum of the right hind paw of BALB/c mice under anesthesia (ketamine 50 mg/kg plus xylazine 5 mg/kg).

Once the lesions emerged (∼4 weeks), the animals were randomly separated into four groups and treated as follows: Group 1—Meglumine antimoniated via intraperitoneal (I.P.) injection and topical ozonated oil group (MTO_Z_); Group 2—Topical ozonated oil group (TOz); Group 3—meglumine antimoniate I.P. group (M); and Group 4—Untreated group (U).

#### Treatment Protocols

The treatments were initiated after the appearance of paw lesions (approximately 8 weeks after parasite inoculation), and the animals received ozonated or nonozonated oil and meglumine antimoniate (MA) daily for 30 days. The groups subjected to sunflower oil application received 0.1 mL of oil topically via a swab, whereas the groups subjected to MA treatment received the drug I.P. at a dose of 50 mg/kg.

#### Lesion Measurements

Measurements and photographic records of the paws were taken by an operator blind to the groups. Besides, all of the analyses after the euthanasia were conducted by blinded operators to the groups. Paw length (mm) was measured via a digital caliper once a week during the treatment period. Photographic records were obtained to monitor the degree of healing of the lesion.

#### Blood Leukocyte Count and Differential Analysis

Before the beginning and after the treatment period, blood leukocytes were collected and subjected to counting and differential analysis. The blood was obtained from a cut in the tail, and 5 μL was added to 45 μL of Turk solution; the cells were then counted in a Neubauer chamber. Another blood aliquot was collected, and a blood smear was performed on a glass slide and stained with a Giemsa stain.

#### Lesion Analysis

After 30 days of treatment, the mice were euthanized via anesthetics (ketamine 270 mg/kg and xylazine 30 mg/kg). The injured paws were removed and divided into four segments: one was placed in culture to observe the development of viable forms of *L. amazonensis*; the second was subjected to Western blot (WB) analysis; the third segment was subjected to tissue oxidation analysis; and the fourth was analyzed via a histological technique. The techniques are explained below.

#### Parasite Quantification in Lesion Culture

The parasite quantification was performed as described by Pivotto et al.^31^ Briefly, the fragment of the lesion was removed aseptically and placed in a microtube containing 199 mediums previously weighed. After fragment addition, the tube was weighed again. The fragment was subsequently macerated with a syringe plunger. Then, the contents were carefully passed into a 1 mL syringe approximately 20 times, after which the contents received another 2 mL of Medium 199. In a 96-well plate, a serial dilution was made in titers ranging from 1/3 to 1/384. The plate was incubated in B.O.D. at 25 °C and the cultures were observed for 5 days via an inverted microscope, evaluating the presence of viable promastigote forms of *L. amazonensis*. All titrations that showed parasites were verified by withdrawing an aliquot and observing it under a regular microscope.

#### Analysis of ERK 1/2 Expression by Western Blot

The segment subjected to analysis by WB was frozen in liquid nitrogen until protein extraction. The tissue was processed in polytron equipment until complete homogenization was achieved via tissue protein extraction buffer (10 mM EDTA, 100 mM Trisma base, sodium pyrophosphate, sodium fluoride, 2 mM PMSF, aprotinin, deionized water, and 10 mM Triton). The mix was placed on ice for 40 min and then centrifuged at 10,000 rpm for 40 min. After centrifugation, the supernatant (protein extract) was used for protein determination via the Bradford method.^32^ For the WB analysis, the lysates were subjected to SDS–PAGE (10%). Protein separation was performed in an electrophoresis tank (running buffer: 25 mM Tris; 192 mM glycine; 0.5% SDS) at 125 V for approximately 120 min. The proteins were transferred to a nitrocellulose membrane via transfer buffer (25 mM Tris; 192 mM glycine; 20% methanol) at 90 mA for 90 min. The nitrocellulose membrane was subsequently placed in a nonfat milk solution (5%) in Tris-Saline-Tween buffer (TBST) for 1 h to minimize nonspecific binding, incubated with primary antibody at a dilution of 1:1000 for 15 h, and stirred at 4 °C. The antibody was removed, and the membranes were washed and incubated with a secondary antibody for 2 h (dilution 1:20,000). At each incubation step, the membrane was washed with TBST for 5 min three consecutive times. Antimurine ERK 42/44 phosphorylated (obtained from rabbit) (Cell Signaling Technology, Denver, Massachusetts) was used as the primary antibody. The antimurine GAPDH antibody (obtained from rabbit) (Cell Signaling Technology, Denver, Massachusetts) was used to normalize the values acquired with the primary antibodies, and as a secondary antibody, HRP-conjugated antirabbit IgG (obtained from mouse) (Abcam, Cambridge, UK) was used. Visualization was performed via enhanced chemiluminescence (ECL; Amersham, Arlington Heights, Illinois) with Chemi L-Pix Express photodocumenting equipment (Locus Biotecnologia, São Paulo, São Paulo, Brazil). The relative band density was analyzed via Image Studio Lite, Version 5.2 software.

#### Biomarkers of Antioxidant Activity

The fragments subjected to the activity of the antioxidant enzymes were frozen in liquid nitrogen until protein extraction. The paw fragments were macerated in phosphate buffer (pH 7.4) and centrifuged at 12,000 rpm for 10 min at 4 °C. The protein concentration was determined using the Bradford method, with a bovine albumin curve.^32^ The supernatant was used in the analyses: Glutathione peroxidase, glutathione S-transferase activity, and lipid peroxidation were measured as described by da Silva Scarton et al.^33^

#### Collagen Quantification

The fragments subjected to histological analysis were fixed in 10% neutral-buffered formalin, decalcified in 5% trichloroacetic acid, routinely processed for paraffin embedding, and stained with picrosirius red for quantification of total collagen. To perform morphometric collagen analysis, images were obtained from five fields of five slides from each animal at a final magnification of 400x under polarized light. The images were digitized through an Olympus BX43 with a DP71 camera, and morphometric quantification was performed on digital images via the software GIMP. A single observer blinded to the conditions and treatments performed the analysis.

#### Obtaining Peritoneal Macrophages

To obtain peritoneal macrophages from the animals, peritoneal lavage was used. 10 mL of ice-cold PBS was added to the exposed peritoneum, and the cells were detached through massage. Afterward, with the same needle and syringe used for inoculation, the peritoneal lavage was collected, and this material was centrifuged at 1500 rpm for 6 min at 4 °C.

The pellet was resuspended in 1 mL of RPMI with 10% FSB. The concentration was altered to 2 × 10^5^ cells/well (200 μL), and the cells were plated in triplicate in a 96-well plate (37 °C; 5% CO_2_) and incubated for 2 h. The supernatant was subsequently collected and substituted with fresh RPMI medium. The cells were incubated for 48 h under the same conditions, after which the supernatant was obtained and used to measure the NO and cytokine (TNF-α, IL-10, and IL-6) levels.

#### Obtaining Popliteal Lymph Nodes

The lymph nodes draining the lesions were removed aseptically from the mice, and the homogenate was prepared as described in the Parasite Quantification in Lesion Culture section. The cell concentration was adjusted to 2 × 10^6^ cells/well (400 μL) in a 24-well plate and incubated for 48 h (37 °C; 5% CO_2_), and the medium was subsequently collected to measure the NO and cytokine levels. NO was measured on the day of collection. The supernatants used for the detection of the cytokines were stored at −80 °C until measurement.

### Measurement of Nitric Oxide (NO) Levels

The NO concentration was measured by determining the nitrite levels in the medium collected after 48 h of culture, as described by Green et al., with some modifications.^34^ 100 μL of the supernatant was mixed with 100 μL of Griess reagent (0.1% (w/v) naphthyl ethylenediamine in 5% (v/v) ortho-phosphoric acid) and 1% (w/v) *p*-aminobenzenesulfonamide in 5% (v/v) phosphoric acid. After the compound was stabilized (10 min), the plate was read at 550 nm. The data are presented in micromolar (μM) values obtained from a standard curve of sodium nitrite (NaNO_2_) in RPMI medium.

### Cytokine Levels

The cytokines TNF-α, IL-6, and IL-10 produced by peritoneal macrophages and the IFN-γ and IL-4 produced by lymph node cells were detected via enzyme-linked immunosorbent assay (ELISA) via kits from Peprotech, Inc. (New Jersey), following the manufacturer’s instructions.

### Statistical Analysis

The data were analyzed via one-way ANOVA with Tukey’s post hoc test (confidence level of 95% and *p* < 0.05). The analyses were conducted with GraphPad Prism, version 6.0, and Microsoft Excel, both of which are Microsoft’s Windows 10 platform.

## Supporting Information Available

Ozone concentration in oil; photographs of lesions in animals; photomicrographs of lesions dyed by picrosirius red; and differential cell blood counting (PDF)
