# Supplementary material for: Ozonated Sunflower Oil Stimulates the Local Antioxidant System and Helps Meglumine Antimoniate to Ameliorate Cutaneous Leishmaniasis Lesions
Source: ACS Omega. 2025 Apr 22;10(17):17543–50. doi: 10.1021/acsomega.4c11263 (PMC12059952; doi:10.1021/acsomega.4c11263)
Supplement: Supplementary file 1 — ao4c11263_si_001.pdf [file ao4c11263_si_001.pdf]

Ozonated sunflower oil stimulates the local antioxidant system and helps meglumine antimoniate to ameliorate cutaneous leishmaniasis lesions

Isaac Loreiro Cabral[1], Lucas Bonatto de Souza Lima[1], Daniela Patrícia Três[1], Carla Diel Fabrini[1], Gislayni Carolini da Silva[1], Camilla Zottesso Pellon Ferreira[1], Fernanda Coleraus Silva[2], João Paulo de Arruda Amorim[2], Thaís Soprani Ayala[1], Rafael Andrade Menolli[1*]

[1] Laboratory of Applied Immunology, Center of Medical and Pharmaceutical Sciences, Western Parana State University, 2069 Universitaria st, Jd. Universitario, 85819-110, Cascavel/PR, Brazil

[2] Center of Biological and Health Sciences, Western Parana State University, 2069 Universitaria st, Jd. Universitario, 85819-110, Cascavel/PR, Brazil

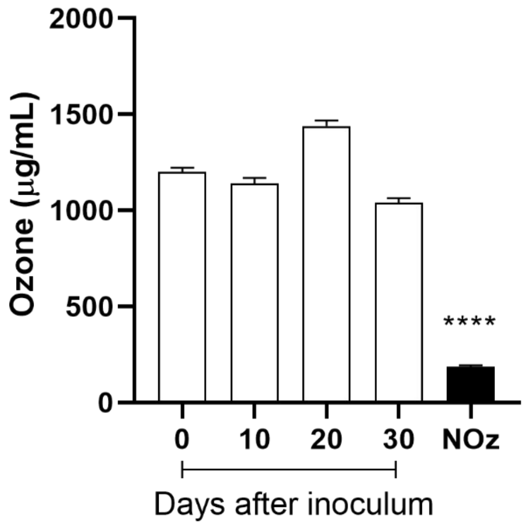

Supplementary figure 1 – Ozone concentration in the sunflower oil ozonated for 15 hours. The levels were detected immediately after the ozonization and 10, 20, and 30 days. The non-ozonated sunflower oil (NOz) ozone levels were determined on day 0. *Significantly different from the other groups (p<0.05). The data are presented as the means of three measurements (±SEM) and were analysed through ANOVA with Dunnett's post hoc test, with a confidence level of 95%.

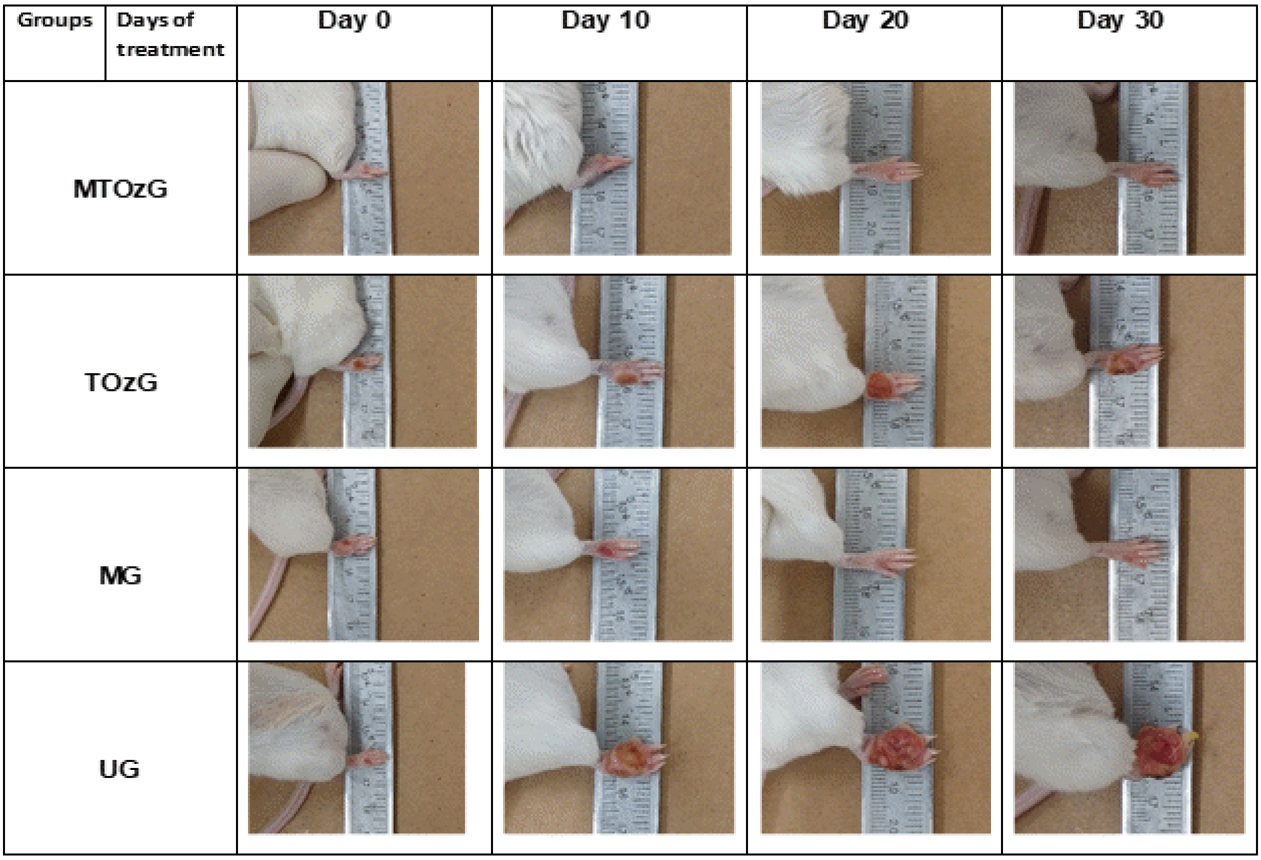

Supplementary figure 2: Lesions aspects of the paws from mice infected with *L. amazonensis*, followed during 30 days of treatment. Pictures of one mouse per group were randomly selected, following the evolution of lesions, and photographed at days 0, 10, 20, and 30. MTOzG - Meglumine antimoniated IP and topical ozonated oil group; TOzG - Topical ozonated oil group; MG – Meglumine antimoniate IP group and UG - Untreated group.

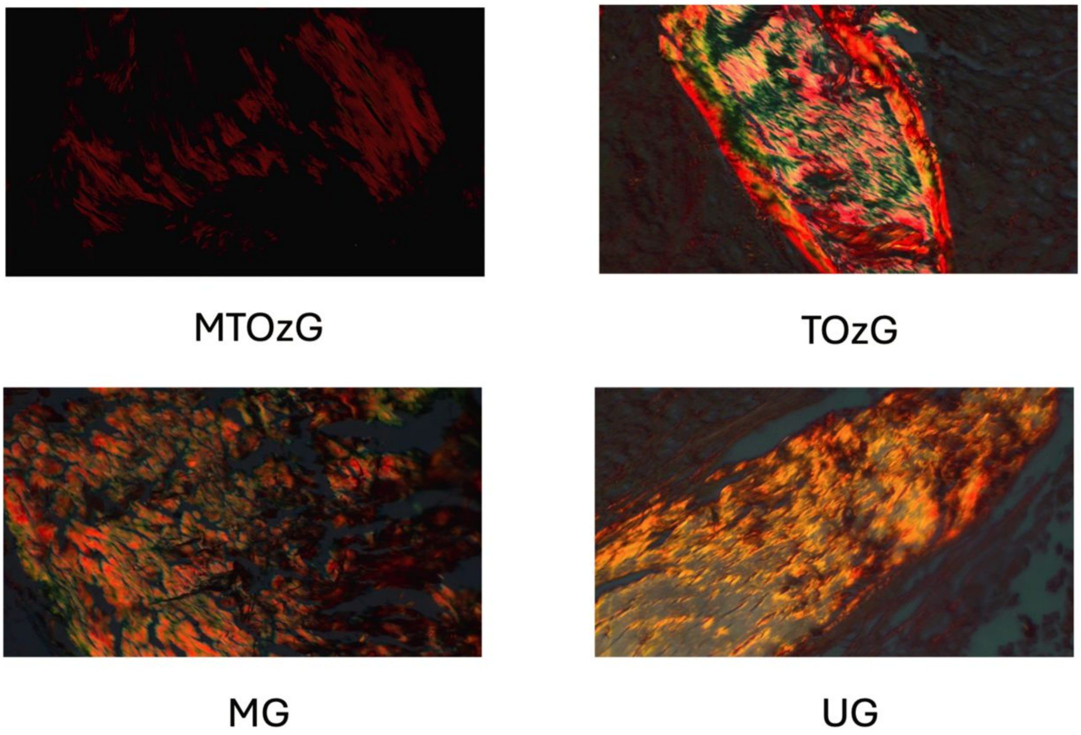

Supplementary figure 3 – Representative photomicrographs of sections from lesions of the paws from mice infected with *L. amazonensis* stained by picrosirius-red and obtained with polarized light, after 30 days of treatment. MTOzG - Meglumine antimoniated IP and topical ozonated oil group; TOzG - Topical ozonated oil group; MG – Meglumine antimoniate IP group and UG - Untreated group.

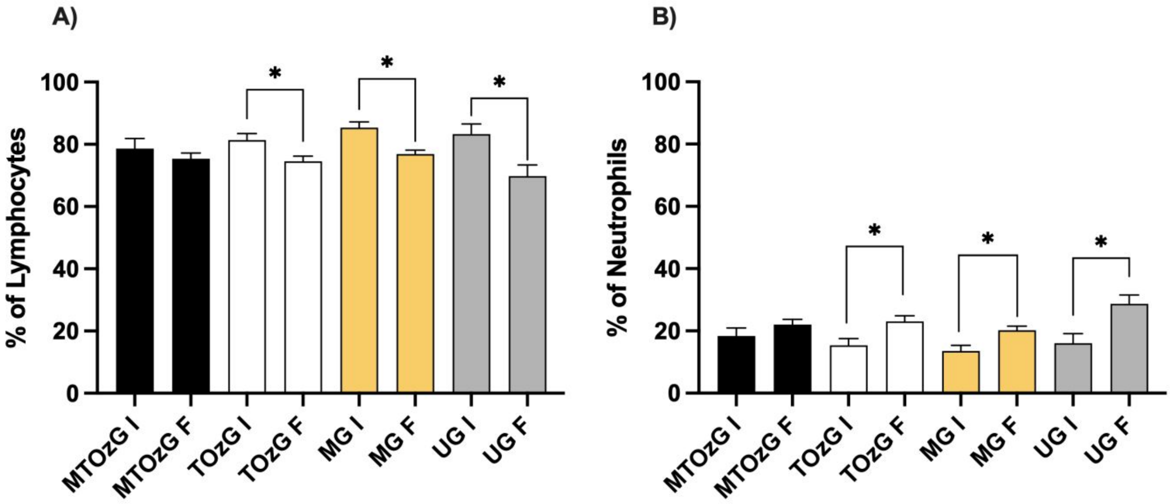

Supplementary figure 4 – Differential blood leucocyte count of (A) lymphocytes and (B) neutrophils of animals infected with *L. amazonensis* after 30 days of treatment. MTOzG - Meglumine antimoniate IP and topical ozonated oil group; TOzG - Topical ozonated oil group; MG – Meglumine antimoniate IP group and UG - Untreated group. The letters I and F in the X axis means Initial and Final (I – before the treatments and F – after 30 days of treatment). The values shown are the mean±SEM of four to six experimental units. *Significantly different (*$p<0.05$). The data were analysed through Unpaired t test, with a confidence level of 95%.
